# Vitexin reduces epilepsy after hypoxic ischemia in the neonatal brain via inhibition of NKCC1

**DOI:** 10.1186/s12974-018-1221-6

**Published:** 2018-06-20

**Authors:** Wen-di Luo, Jia-wei Min, Wen-Xian Huang, Xin Wang, Yuan-yuan Peng, Song Han, Jun Yin, Wan-Hong Liu, Xiao-Hua He, Bi-Wen Peng

**Affiliations:** 1 Department of Physiology, Hubei Provincial Key Laboratory of Developmentally Originated Disorder, School of Basic Medical Sciences, Wuhan University, Hubei Donghu Rd 185#, Wuhan, 430071 Hubei China; 2 Department of Pathology, Renmin Hospital, Wuhan University, Wuhan, China; 3 Department of Pathophysiology, School of Basic Medical Sciences, Wuhan University, Wuhan, China; 4 Department of Immunology, School of Basic Medical Sciences, Wuhan University, Wuhan, China

**Keywords:** HIBD, Vitexin, NKCC1, F-actin, Blood-brain barrier, Inflammation, Epilepsy

## Abstract

**Background:**

Neonatal hypoxic-ischemic brain damage, characterized by tissue loss and neurologic dysfunction, is a leading cause of mortality and a devastating disease of the central nervous system. We have previously shown that vitexin has been attributed various medicinal properties and has been demonstrated to have neuroprotective roles in neonatal brain injury models. In the present study, we continued to reinforce and validate the basic understanding of vitexin (45 mg/kg) as a potential treatment for epilepsy and explored its possible underlying mechanisms.

**Methods:**

P7 Sprague-Dawley (SD) rats that underwent right common carotid artery ligation and rat brain microvascular endothelial cells (RBMECs) were used for the assessment of Na^+^-K^+^-Cl^−^ co-transporter1 (NKCC1) expression, BBB permeability, cytokine expression, and neutrophil infiltration by western blot, q-PCR, flow cytometry (FCM), and immunofluorescence respectively. Furthermore, brain electrical activity in freely moving rats was recorded by electroencephalography (EEG).

**Results:**

Our data showed that NKCC1 expression was attenuated in vitexin-treated rats compared to the expression in the HI group in vivo. Oxygen glucose deprivation/reoxygenation (OGD) was performed on RBMECs to explore the role of NKCC1 and F-actin in cytoskeleton formation with confocal microscopy, *N*-(ethoxycarbonylmethyl)-6-methoxyquinolinium bromide, and FCM. Concomitantly, treatment with vitexin effectively alleviated OGD-induced NKCC1 expression, which downregulated F-actin expression in RBMECs. In addition, vitexin significantly ameliorated BBB leakage and rescued the expression of tight junction-related protein ZO-1. Furthermore, inflammatory cytokine and neutrophil infiltration were concurrently and progressively downregulated with decreasing BBB permeability in rats. Vitexin also significantly suppressed brain electrical activity in neonatal rats.

**Conclusions:**

Taken together, these results confirmed that vitexin effectively alleviates epilepsy susceptibility through inhibition of inflammation along with improved BBB integrity. Our study provides a strong rationale for the further development of vitexin as a promising therapeutic candidate treatment for epilepsy in the immature brain.

## Background

Perinatal hypoxic-ischemic encephalopathy (HIE) secondary to perinatal asphyxia remains a major cause of neonatal mortality and is associated with long-term neurologic comorbidities both in the late preterm and term neonate [1]. Approximately 15 to 25% of newborns die in the postnatal period, and 25% develop severe and permanent neuropsychological sequelae, including cerebral palsy, visual impairment, mental retardation, learning impairment, and epilepsy [2–5]. It has also been reported that HIE is one of the most common devastating etiologies associated with neonatal seizures, constituting 50–60% of the total number of seizures reported in neonates [6, 7]. However, HIE-associated neonatal seizures are often unresponsive to conventional antiepileptic drugs (AEDs), and the seizures cannot be controlled in 30% of children due to drug resistance [8, 9]. In the past decade, the mainstay of treatment for HIE remains comprehensive treatment, including hyperbaric oxygen therapy and mild hypothermia therapy, and medication. Although this therapy has been shown to improve survival and neurodevelopmental outcome, the neuroprotective response is limited by timing of initiation and severity of encephalopathy [10]. Thus, it is an urgent need to develop effective agents that target vulnerable periods in HIE-induced neonatal epilepsy.

The blood-brain barrier (BBB) plays an important role in brain damage. BBB dysfunction facilitates the infiltration of inflammatory factors and neutrophils, which contribute to morbidity in multiple sclerosis, encephalitis, traumatic brain injury, neurodegenerative diseases, brain tumors, and ischemic and hemorrhagic stroke [11]. BBB leakage occurs after the initial brain insult and is postulated to trigger epileptogenesis by permitting infiltration of inflammatory molecules into the brain [12–15]. Furthermore, inflammation in brain tissue has been fully described in human epilepsy of various etiologies and in experimental rodents with seizures [16–18], indicating that epilepsy could be a cause of the inflammatory response and endothelium impairments [14].

The Na-K-Cl co-transporter (NKCC), which consist of two isoforms (NKCC1 and NKCC2), is a critical transmembrane protein family playing an important role in cellular ion homeostasis and the subsequent accumulation of intracellular water [19, 20]. And NKCC is one of the major pathways to transport Cl^–^ into cells [21]. NKCC1 is a member of the critical transmembrane protein family, which plays an important role in maintaining CNS functions, such as cell migration, astrocyte swelling [22], and neuroblast migration [23]. NKCC1 expression is developmentally regulated in the human and rodent brain, peaking in neurons, astrocytes, and oligodendrocytes in early perinatal development before declining in adulthood [20, 22, 24–26]. In addition to acting as an ion transporter, NKCC1 also participates in interactions with the actin cytoskeleton, and there is decreased expression of F-actin content upon NKCC1 knockdown [27, 28]. F-actin stress fibers present in injured endothelial cells, which leads to endothelial contraction and dismantlement of ZO-1, which maintains the integrity of the BBB [29]. The proposed ZO-1-regulatory mechanisms that are affected by F-actin may be based on the unique C-terminal half of ZO-1, which co-precipitates with F-actin. In contrast, the construct encoding the N-terminal half of ZO-1 is specifically associated with tight junctions (TJs) [30]. It has also been well documented that inhibition of NKCC1 may protect against traumatic brain injury-induced BBB breakdown [26]. Moreover, an increase in NKCC1 in neurons partially elevates excitability in neonatal seizures [25, 31], and the pharmacomodulation of chloride co-transporters has been investigated in treating refractory neonatal seizures [32–34].

Vitexin, which is a naturally derived flavonoid compound found in many medicinal plants, has recently received increasing attention due to its numerous pharmacological properties. Previous studies have shown that in vivo and in vitro treatment of flavonoid exerts protective effects by reducing pro-inflammatory cytokine secretion and restoring the levels of tight junction proteins of the BBB [35–38]. For instance, vitexin possesses a cardio-protective effect, which may be associated with its anti-oxidative effects, activation of ER stress, and inhibition of inflammatory cytokine release [39–41]. Vitexin also attenuated neuronal death and reduced neonatal brain injury in rats induced by hypoxia-ischemia via rescuing BBB collapse [42]. In addition, vitexin provides short- and long-term neuroprotection in pilocarpine-induced seizures in rats and exerts antiepileptic activity and neuroprotection [43]. Investigations of the underlying mechanism of action have come to the assumption that vitexin seems to be a ligand for benzodiazepine receptor, which could allosterically modulate GABA_A_ receptors by binding to the benzodiazepine receptor site [44–46].

Nevertheless, although there have been a few studies demonstrating the antiepileptic activity of vitexin using in vivo and in vitro experimental models, none of these investigated its potential mechanism involved in NKCC1. The present study firstly showed that vitexin is beneficial to the treatment of HI insult through disturbing the NKCC1/F-actin pathway, which potentially protects against the severity of BBB collapse. Therefore, this study was designed to investigate the effect of vitexin in alleviating seizures during neonatal brain damage through preserving the integrity of the BBB via inhibition of NKCC1.

## Methods

### Experimental animals and groups

Sprague-Dawley (SD) rats (postnatal day 7) were provided by the Animal Biosafety Level 3 Laboratory (ABSL-3, Wuhan University, China). All rats were housed at the standard laboratory animal facility (25 ± 2 °C, 12-h light/dark cycle) with access to food and water ad libitum in individual cages.

The rats (*n* = 128) were randomly assigned to four groups (*n* = 32 rats each): hypoxia-ischemia group (abbreviated: HI group), rats underwent the ligation of the right common carotid artery and had hypoxic injury, the details of which are discussed in the following experimental methods; sham-operated group (abbreviated: sham group), rats underwent the same operation, without ligation of the right common carotid artery nor the hypoxia treatment; HI+bumetanide group (abbreviated: HI+Bum group), bumetanide (0.5 mg/kg, intraperitoneal injection, i.p., Cat. sc-200727, Santa Cruz) was diluted with saline and administered 5 min after HI; bumetanide, an inhibitor of NKCC1, served as positive group in our study. HI+vitexin group (abbreviated: HI+Vit group), vitexin (45 mg/kg, intraperitoneal injection, i.p., Cat. E-0310, Tauto Biotech) was diluted with saline and administered 5 min after HI.

### Neonatal hypoxia-ischemia model

All procedures of the neonatal HI model in this study were based on the Rice-Vanucci study [47, 48]. Animal surgical procedures and experimental protocols were reviewed and approved by the Committee on the Ethics of Animal Experiments of Wuhan University (China). Briefly, P7 SD rats (13–19 g, equal males and females have been chosen for each group) were anesthetized by inhalation of isoflurane. The right common carotid artery (CCA) was exposed and ligated with 5-0 surgical silk. After ligation for 2 h, the rat pups were then placed in an airtight, transparent chamber at 37 °C and exposed to 8% O_2_ in N_2_ for 3 h to create hypoxic injury after brain ischemia. Thereafter, the littermates were returned to their mothers when they can move freely. Meanwhile, sham-operated rats were subjected to isolation and stringing of vessels without ligation and subsequent ischemia. The rats were sacrificed at 24 h after HI insult, and their ipsilateral hemispheres were collected for follow-up experiments.

### Cell culture and oxygen glucose deprivation (OGD) progression

Lines of rat brain microvascular endothelial cells (RBMECs) were purchased from BeNa Culture Collection (Cat. BNCC337880). RBMEC cultures were expanded and maintained in 89% basal medium, 10% fetal bovine serum (FBS), and 1% penicillin/streptomycin solution (P/S). They were then incubated in a humidified atmosphere containing 5% CO_2_ at 37 °C.

OGD was conducted as described previously. Briefly, RBMECs were grown in complete growth media as monolayers in cell culture incubator (95% O_2_ and 5% CO_2_ at 37 °C). To initiate OGD in vitro, the cells at 4 DIV (days in vitro) were washed with 1× PBS three times, switched to OGD medium (serum- and glucose-free DMEM), and placed in a hypoxic/anoxic chamber (1% O_2_, 5% CO_2_, and 94% N_2_ at 37 °C) to mimic OGD injury in incubator. Following the OGD carried out for 3.5 h, cells were removed from the anaerobic chamber, and the OGD medium in the cultures was then changed back to DMEM medium for an additional 24-h reperfusion under normal conditions. At the same time, control glucose-containing cultures remained in a regular incubator (5% CO_2_ and 95% O_2_). The supernatants and cell extracts were collected after OGD for the following experiments.

### Protein isolation and western blot

Membrane protein was extracted according to the manufacturer’s protocol from cultured RBMECs and the ischemic penumbra of the rat cortex with a Membrane and Cytosol Protein Extraction Kit (Cat. P0033, Beyotime, Shanghai, China). The concentrations were determined with a BCA protein assay kit (Cat. P0012, Beyotime, Shanghai, China). Samples were denatured for 10 min at 100 °C and frozen at − 20 °C before assay. Approximately 20 μl of the samples were separated by 8–12% SDS–PAGE gel and then transferred to polyvinylidene fluoride (PVDF) membranes. Subsequently, the membranes were blocked in 5% BSA for 1 h at room temperature following incubation with primary antibodies overnight at 4 °C. Dilutions for primary and secondary antibodies were listed in Table 1. Membranes were washed three times in TBST and specific binding was visualized by ECL reaction. The density of bands was detected using an imaging densitometer (Bio-Rad, Foster City, CA, USA), and the gray value of the bands was quantified using ImageJ Software (version 1.41).

**Table 1:** Antibodies applied for western blot

Antibody	Host	Company	Cat. no.	Dilution	Duration
NKCC1	Goat	Santa Cruz Biotechnology	Sc-21545	1:500	Overnight 4 °C
ZO-1	Rabbit	Invitrogen	RA231621	1:100	Overnight 4 °C
β-actin	Mouse	Protein tech	HRP-60008	1:10000	Overnight 4 °C
Anti-goat IgG-HRP	Rabbit	Protein tech	SA00001-4	1:1000	1 h RT
Anti-rabbit IgG-HRP	Goat	PMK Biotech	PMK-014-090	1:100000	1 h RT

### Real-time PCR

Total RNA was extracted from the ischemic cerebral cortices (*n* = 6 per group) for the detection of NKCC1, IL-1β, IL-6, and TNF mRNA at 24 h after HI using Trizol Reagent (Invitrogen Life Technologies Corporation, USA) according to the manufacturer’s protocol. The quantity of total RNA was measured with a UV spectrophotometer (Biochrom Ltd., UK). Next, reverse transcription was performed using a cDNA synthesis kit (TaKaRa Biotechnology). Briefly, 2 μl of total RNA was combined with 4 μl of 5× Prime Script® Buffer. RNase Free ddH_2_O was added to 20 μl, after which the mixture was heated at 37 °C for 15 min and then 85 °C for 5 s. Quantitative PCR was performed with SYBR-Green premix (Trans Gen Biotech) at the following conditions (denaturing at 95 °C for 10 s, followed by 40 cycles of 95 °C for 5 s and 60 °C for 30 s) and detected by a real-time PCR system (Step One, Applied Biosystems). The expression of target genes was measured in triplicate and normalized to β-actin as an internal control. The ΔΔCt values of each group were analyzed, and mRNA expression levels were normalized to 2−ΔΔCt. Primers are listed in Table 2 (Sangon Biotech, Shanghai, Co., Ltd.).

**Table 2:** Primer sequences applied for q-PCR

Primer	Forward prime 5′-3′	Reverse prime 5′-3′
NKCC1	AGACTTCAACTCAGCCACTGT	CAAGGTCAAACCTCCATCATCA
ZO-1	TTGCCACACTGTGACCCTAA	GTTCACACTGCTTAGTCCAGC
IL-1β	GGAACCCGTGTCTTCCTAAAG	CTGACTTGGCAGAGGACAAAG
IL-6	TAGTCCTTCCTACCCCAATTTCC	TTGGTCCTTAGCCACTACTTC
TNF	CCAACAAGGAGGAGAAGTTCC	CTCTGCTTGGTGGTTTGCTAC
β-actin	GATCAAGATCATTGCTCCTCCTG	AGGGTGTAAAACGCAGCTCA

### Enzyme-linked immunosorbent assay (ELISA)

The protein concentrations of IL-1β, IL-6, and TNF in each group of animals were quantified using an ELISA kit (purchased from 4A Biotech Co., Ltd) according to the manufacturer’s instruction. Absorbance at 450 nm was recorded and the concentration of the target protein was calculated according to the standard curve and normalized against the protein of the samples. Result was expressed as pg/mg protein.

### Immunofluorescence

Immunofluorescence staining was carried out to detect ZO-1 and CD31 expression in peri-ischemic brain tissue or ZO-1, NKCC1, and F-actin expression in RBMECs.

Rats in each group were deeply anesthetized with 10% chloral hydrate and transcardially perfused first with PBS and then fixed in 4% paraformaldehyde solution at room temperature, dehydrated, and embedded in paraffin at 24 h after HI. Post-fixation, the brains were removed and cryoprotected in 20% sucrose and 30% sucrose solutions and dehydrated by 30% sucrose for 72 h at 4 °C. Serial coronal sections (5-μm-thick with injury epicenter located centrally) prepared with cryotome (Leica, Wetzlar, Germany) were used for immunofluorescence labeling. Sections were incubated with a blocking solution (5% FBS) for 30 min at 37 °C. The tissue slices were then incubated overnight with the anti-ZO-1 and anti-CD31 antibodies. On the following day, the sections protected from light were washed and subsequently incubated with secondary antibodies Cy3-conjugated Goat Anti-Rabbit IgG (H+L) and Alexa Fluor® 488 Conjugates for 2 h at 37 °C. Dilutions for antibodies were listed in Table 3. Images were obtained using a confocal microscope (Leica-LCS-SP8-STED).

**Table 3:** Antibodies applied for flow cytometry, immunohistochemistry, and fluorescence staining

Antibody	Host	Company	Cat. no.	Dilution	Applied	Stored
NKCC1	Goat	Santa Cruz	Sc-21545	1:50	ICC,FCM,IHC	Overnight 4 °C
NeuN	Mouse	Millipore	#2742283	1:100	ICC	Overnight 4 °C
Phalloidin		Sigma	P5282	5 μg/ml	ICC	1 h RT
Alexa Fluor® 488	Mouse	Cell Signaling Technology	#4408	1:250	ICC	1 h RT
Anti-goat IgG-Cy3	Rabbit	Protein tech	SA00009-4	1:50	ICC	1 h RT
Anti-rabbit IgG-Cy3	Goat	Protein tech	SA00009-2	1:50	ICC	1 h RT
ZO-1	Rabbit/IgG	Invitrogen	RA231621	0.25 mg/ml	ICC,IF	Overnight 4 °C
MPO	Rabbit	Abcam	ab9535	1:100	IHC	Overnight 4 °C
CD31	Mouse	Thermo Fisher Scientific	MA3100	1:50	IF	Overnight 4 °C
Anti-rabbit IgG	Goat	PMK Biotech	PMK-014-090	1:100000	IHC	1 h RT
F-actin	Mouse	Abcam	ab205	1:100	ICC	Overnight 4 °C
Anti-goat IgG-FITC	Rabbit	Protein tech	SA00003-4	1:50	ICC	1 h RT
DAPI		Beyotime	C1002	1:2000	ICC,IF,IHC	1 min RT

For the assessment of ZO-1, NKCC1, and F-actin expression in RBMECs, cells were fixed with methanol, washed with PBS-T, and incubated at 4 °C with anti-NKCC1, anti-F-actin, and anti-ZO-1 antibodies. Subsequently, cells were washed with PBS-T before incubation with mixtures of secondary antibodies: Alexa Fluor® 488 Conjugates, Cy3-conjugated Rabbit Anti-Goat IgG (H+L), and Cy3-conjugated Goat Anti-Rabbit IgG (H+L) diluted in blocking buffer for 2 h in the dark at room temperature. Dilutions for antibodies were also listed in Table 3. The cells were washed three times in PBS-T before they were mounted using DAPI. Finally, cellular co-localization was captured using confocal microscope (Leica-LCS-SP8-STED).

### Immunohistochemistry

To evaluate the expression of NKCC1and the severity of HI-induced inflammation, the slices from paraffin-embedded tissues were subjected to immunohistochemical staining for NKCC1 and myeloperoxidase (MPO), respectively. MPO is a representative marker of neutrophils and is an important index for evaluating the severity of inflammation. It also reflects the extent of inflammation in brain tissue [49]. The sections were washed with PBS and blocked in 5% BSA for 2 h. Thereafter, the primary antibody goat anti-NKCC and rabbit anti-MPO polyclonal antibody were applied. After being rinsed with PBS, the sections were incubated with corresponding secondary antibodies, and nuclei were stained with DAPI. Images were obtained using a confocal laser-scanning microscope (Leica-LCS-SP8-STED).

### Phalloidin staining

For visualization of cytoskeleton F-actin, the cultured RBMECs were processed for direct confocal imaging with FITC-conjugated phalloidin (Cat. P5282, Sigma), which specifically combines with F-actin. Monolayers were rinsed with PBS solution, fixed with 4% paraformaldehyde for 30 min at 4 °C, and permeabilized with 0.3% Triton X-100 for 30 min. The cells were incubated with FITC-phalloidin (5 μg/ml, Sigma) for 1 h at room temperature in the dark. Cells were then counterstained with DAPI for nuclear labelling. Visualization was performed with a Leica-LCS-SP8-STED confocal microscope (Leica Microsystems).

### Measurement of intracellular Cl^−^ concentration ([Cl^−^]_*i*_)

*N*-(ethoxycarbonylmethyl)-6-methoxyquinolinium bromide (MQAE), a chloride-sensitive fluorescent indicator inversely related to intracellular chloride ion concentration, was used to detect [Cl^−^]_*i*_ as previously described [50]. This dye detects the ion via diffusion-limited collisional quenching. The cultured RBMECs were then incubated with 10 mM MQAE (Cat. E3101, Invitrogen) in a Kreb HEPES-buffered isotonic solution [DMEM, 0.1% BSA, 10 mM 4-(2-hydroxyethyl)-1 piperazine-ethanesulfonic acid (HEPES), pH 7.5] for 1 h at 37 °C in the dark. Subsequently, cells were washed with DMEM three times. Fluorescence was excited every 60 s at 340 nm, and emission fluorescence at 460 nm was recorded. Images were collected and analyzed with the Image-Pro Plus 6.0 image-processing software.

### Flow cytometry

The number of NKCC1^+^ RBMECs was analyzed by flow cytometry. Single-cell suspensions were harvested at 24 h after OGD and flushed with PBS. The suspensions were then stained with fluorescently labeled antibodies, anti-NKCC1 antibody, and FITC rabbit anti-goat antibodies. The staining was performed according to the manufacturer’s instructions. Flow cytometric analysis was performed using Flow Jo (version 9.2; Tree Star Inc.).

### Electrode implantation and EEG recording

For the recording of the electrical activity of the brain, electrodes were implanted in rats (*n* = 15 per group) on P28. The rats were anesthetized with 10% chloral hydrate (3 ml/kg) and then fixed into the stereotaxic apparatus. The electrodes were bipolar twisted silver steel and embedded in the skull with dental cement. These electrodes were implanted into the bilateral hippocampal CA3 (3.5 mm posterior to bregma, 3.5 mm lateral, 3.5 mm ventral to the dura mater), according to the coordinates derived from the atlas of Paxinos and Watson. Spontaneous EEG seizures in the dentate gyrus were recorded in freely moving animals after 3 days of recovery, which were then individually placed in a cage and connected to a neurophysiology workstation (AD Instruments Lab Chart 8) through a flexible cable that prevents twisting [51]. The frequency and mean duration of these spontaneous EEG seizures during an EEG recording session were examined for 2 h. The EEG signals were digitized with Lab Chart software (AD Instruments). Seizure severity was classified into five levels by Racine’s scale [52]: I, facial movement; II, head nodding; III, unilateral forelimb clonus; IV, bilateral forelimb clonus; V, tonic clonic seizure, rearing, and failing. The rats in which seizure severity reached a level III were identified as grand mal seizure disorder. Seizures were also identified by consistent changes in the power of the fast Fourier transform of EEG, including changes in the frequency of activity during the course of the event. These criteria have been used successfully by experts in the field [53].

### Statistical analysis

Statistical differences between groups were analyzed with either an unpaired *t* test or one-way analysis of variance (ANOVA) where appropriate. Post hoc analysis was performed with the Newman-Keuls multiple-comparison test. Differences were considered statistically significant at a critical value of *P < 0.05. All values are presented as the mean ± standard error of the mean (SEM).

## Results

### NKCC1 mRNA and protein expression in the peri-ischemic brain tissue

**Fig. 1:**
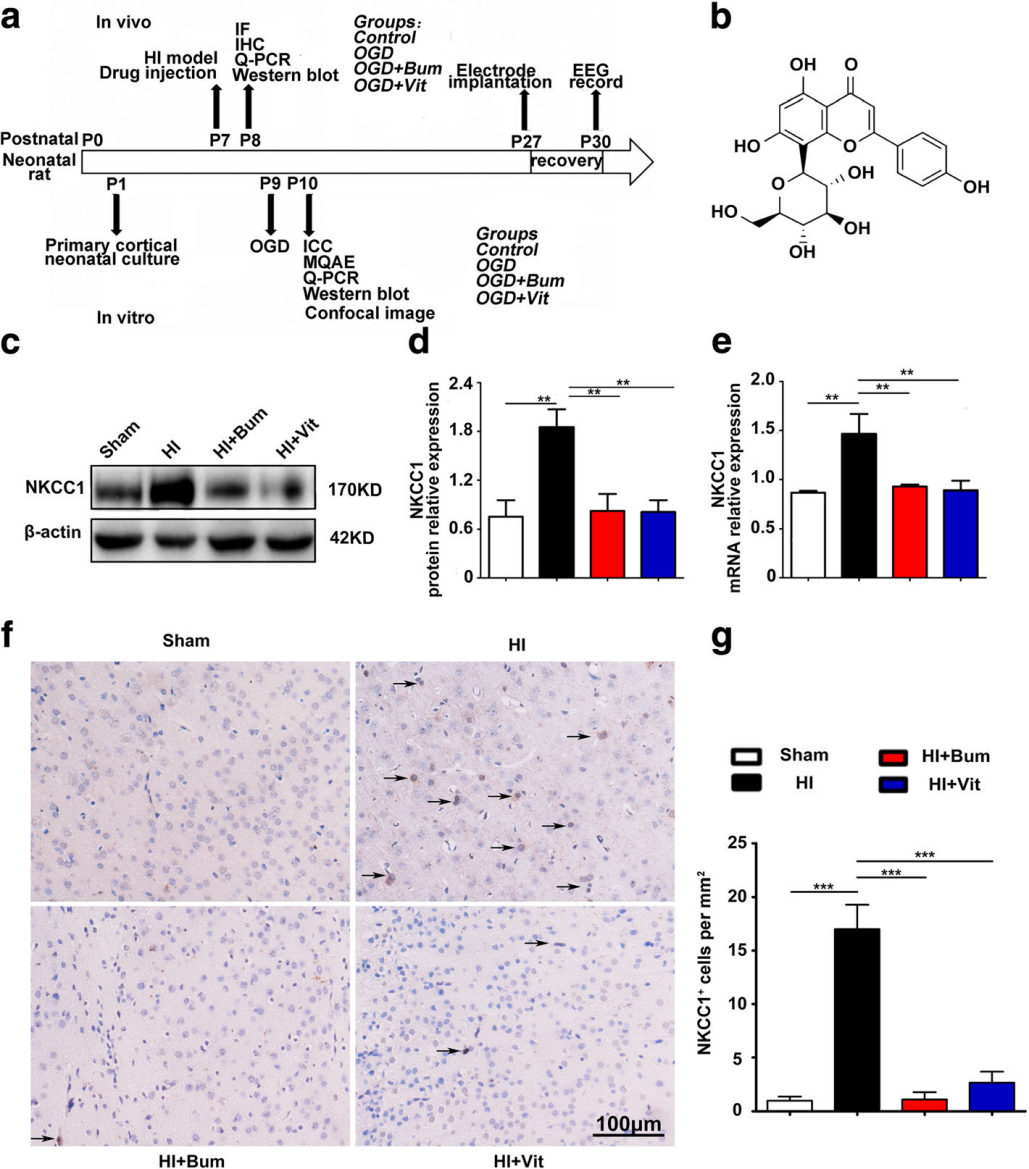
Structure and effect of vitexin on HI-induced NKCC1 expression in hypoxia-ischemia brain tissue. **a** Diagram of the experimental design. **b** The structure of vitexin. **c**, **d** Representative protein expression levels of NKCC1 (170 kDa) and β-actin (42 kDa) in the cerebral tissue of the sham, HI, HI+Bum, and HI+Vit groups were evaluated. **e** The graphical representation of the fold changes in NKCC1 mRNA expression in each group as quantified by normalization to β-actin as an internal control. Data are shown as the mean ± SEM; **P < 0.01 compared to the HI group, *n* = 4 per group, based on a one-way ANOVA. **f** Cortical penumbral regions of coronal sections from the rats of each group were subjected to immunohistochemistry using an anti-NKCC1 antibody, and stereological counts of NKCC1^+^ cells (arrows) in each group are shown. Scale bar = 100 μm. **g** The graph shows the mean number of NKCC1^+^ cells per square millimeter. Data are expressed as the mean ± SEM; one-way ANOVA, ***P < 0.005 in comparison with the HI group, *n* = 4 per group

To determine the profile of NKCC1, we analyzed the protein and mRNA expression of NKCC1 at 24 h following HI. We induced the HI model in P7 neonatal rats, and samples were extracted from the ipsilateral cerebral cortex at 24 h after HI (Fig. 1a). Our results showed an upregulation of the protein and mRNA expression of NKCC1 in the peri-ischemic brain tissue. The optical density of the immune-reactive bands of NKCC1 protein levels that appeared at approximately 170 kDa were significantly increased at 24 h following HI compared with that in the sham group (Fig. 1c). However, after treatment with vitexin, the structure of which is depicted in Fig. 1b, the optical density was decreased significantly when compared with the optical density in ischemic rats (Fig. 1d, *P < 0.05). NKCC1 mRNA expression was significantly increased in the peri-ischemic brain tissue at 24 h following HI rats in comparison with the expression in the sham-operated rats. However, after treatment with bumetanide, which acted as a positive control, or vitexin, the level of NKCC1 mRNA expression in the peri-ischemic brain tissue was significantly decreased when compared with that in the ischemic rats (Fig. 1e, *P < 0.05). Moreover, we performed immunohistochemistry of coronal sections from the ipsilateral cortex to detect NKCC1^+^ cells using anti-NKCC1 antibody (Fig. 1f,  gf, ***P < 0.001). NKCC1^+^ cells were rarely observed in the sham-operated rats, whereas they were more abundant and more extensively distributed in the HI group, particularly in the ischemic penumbra (Fig. 1f, g). After treatment with bumetanide or vitexin, NKCC1^+^ cells in the ipsilateral penumbra were dramatically reduced compared with those in the HI group (Fig. 1f, g). In addition, NKCC1 is located predominantly in the luminal membrane of BBB endothelial cells in situ [54, 55].

### Expression of NKCC1 in RBMECs after OGD treatment

**Fig. 2:**
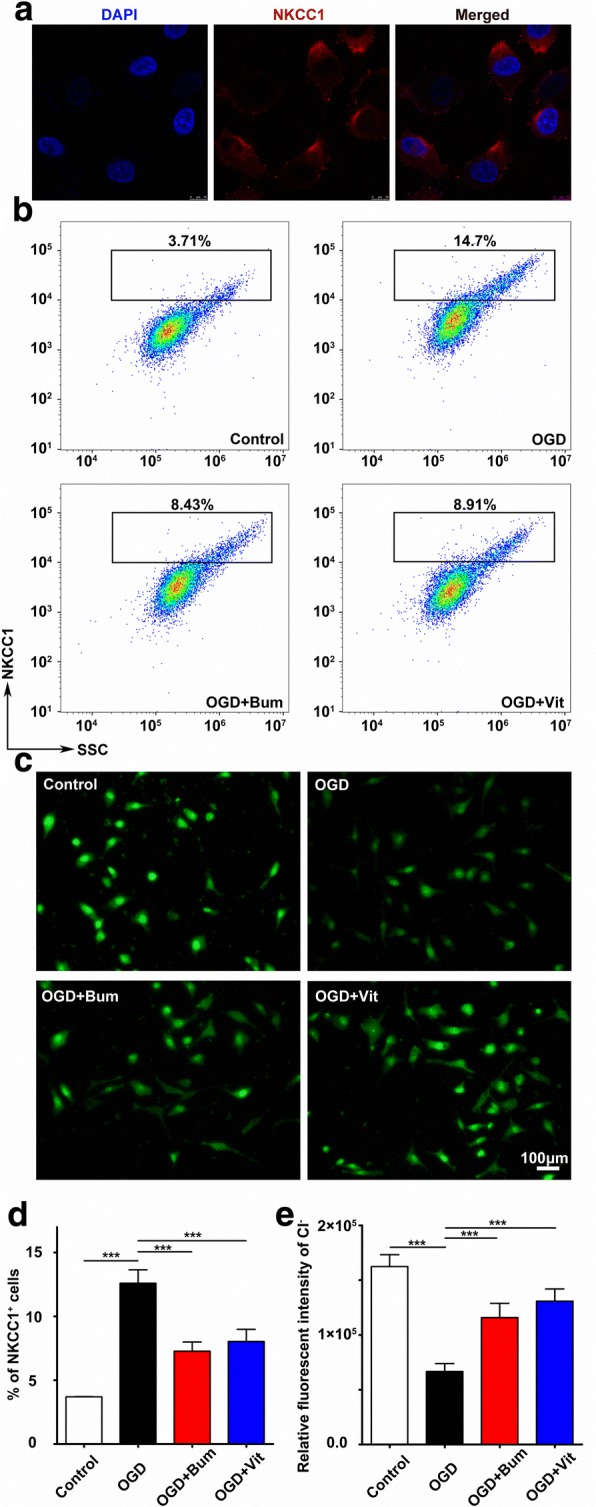
Expression of NKCC1 in RBMECs after OGD. **a** Confocal image demonstrated that NKCC1 expression was localized in RBMECs. RBMECs were fixed and stained with anti-NKCC1 (red) and DAPI-stained nuclei (blue) in the control group, OGD conditions, OGD+Bum group, and OGD+Vit group. Images shown are representative of at least three independent experiments. **b**, **d** Flow cytometry analysis and the quantification of NKCC1-positive cell numbers in RBMECs. Cells were obtained at 24 h after OGD and were subjected to analysis using a flow cytometer after being immunostained with NKCC1 antibodies. Representative results from three to four tests of NKCC1-positive cells from each group are shown. **c**, **e** Fluorescence imaging of Cl^−^ via MQAE stained in RBMECs. The green fluorescence indicated the intracellular level of Cl^−^ in RBMECs and the fluorescence intensity of the controls was high while the RBMECs subjected to OGD were markedly decreased. These changes are readily reversible after treatment of OGD-exposed cells with bumetanide (100 mM; 24 h) or vitexin (100 mM; 24 h). Data are shown as the mean ± SEM; ***P < 0.005 compared to the OGD condition, *n* = 8~10 per group, scale bar = 100 μm, based on a one-way ANOVA

Our previous studies have shown that vitexin has a protective effect on the BBB in HI neonatal brain injury [47]. To further investigate the mechanisms underlying HI-induced BBB disruption, we adopted an in vitro BBB model composed of a monolayer of RBMECs and subjected this model to the ischemia-like insult OGD for 3.5 h [47]. Subsequently, the expression of NKCC1 in RBMECs was confirmed by confocal imaging (Fig. 2a).

To further confirm NKCC1 expression in RBMECs under different conditions, flow cytometry was also used. As shown in Fig. 2b, the number of NKCC1-positive cells increased after OGD compared to that in controls. The cell quantities in both the bumetanide (100 mM; 24 h) and vitexin (100 mM; 24 h) groups were decreased compared to those in the OGD group. In addition, we performed MQAE to analyze the concentration of Cl^−^ in RBMECs (Fig. 2c). This was studied by preloading the cells with the Cl^−^ fluorescent indicator, MQAE, with subsequent analysis of the changes in fluorescence that occurred with changes in the intracellular Cl^−^ levels, which were inversely related to the intracellular chloride ion concentration ([Cl^−^]_*i*_). Compared with the fluorescence intensity in the controls, the fluorescence intensity of RBMECs was significantly decreased in the OGD group, indicating that [Cl^−^]_*i*_ was enhanced following OGD (Fig. 2d). Fluorescence was dramatically decreased with treatment with bumetanide or vitexin. Taken together, these results suggested that vitexin effectively suppresses intracellular [Cl^−^]_*i*_, which indirectly represents the expression of NKCC1.

### Vitexin rapidly reduces actin stress fiber expression in RBMECs after OGD

**Fig. 3:**
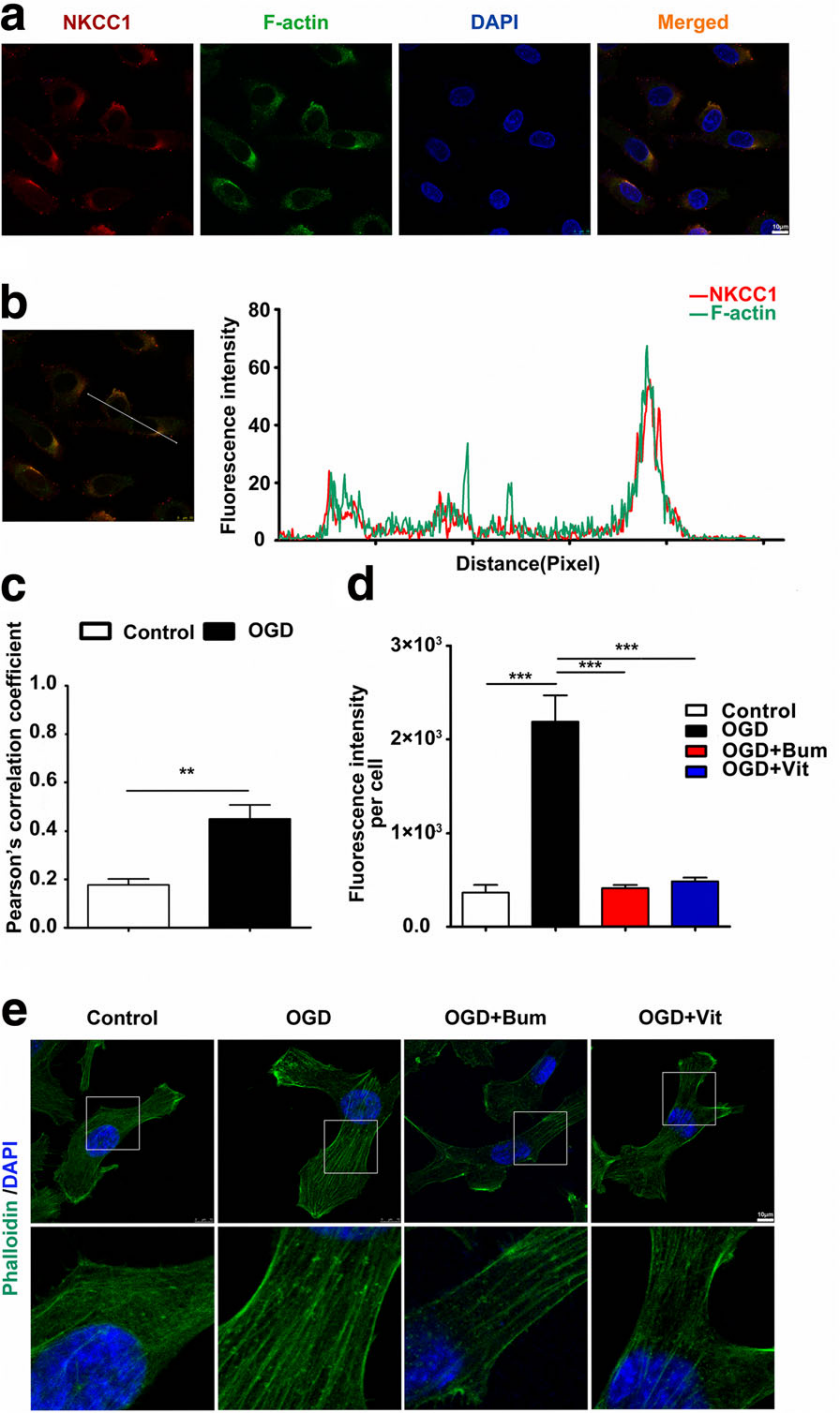
Vitexin downregulated F-actin expression in RBMECs after OGD. FITC-conjugated phalloidin staining for F-actin^+^ stress fiber formation demonstrated changes in cytoskeletal assembly following OGD in RBMECs. **a** Confocal images show co-localization of NKCC1 and F-actin in RBMECs. Scale bar = 10 μm. **b** Merged views of indicated NKCC1 show complete co-localization (**b**, left). A side overlap of two peaks was taken as a partial co-localization (**b**, right). **c** Pearson’s correlation coefficient is shown in graph (**c**) from five independent experiments analyzed. **d**, **e** The distribution of F-actin^+^ stress fibers was shown by confocal imaging in Control, OGD, OGD+Bum, and OGD+Vit groups. Arrows indicate actin stress fiber formation. The intensity of F-actin fluorescence was determined in 10 fields/well and divided by the number of cells counterstained with DAPI. Each experimental group consisted of three replicates. Scale bar = 10 μm

In addition to its conventional function as an ion transporter, NKCC1 also modulates cell migration ability by interacting with the cytoskeleton and acting as an anchor that transduces contractile forces [27, 28]. We therefore examined the NKCC1-positive cells co-stained with the F-actin antibody with confocal microscopy. Our results showed that NKCC1 was localized to F-actin in cultured RBMECs at 24 h following HI (Fig. 3a–c). We also investigated the effects of vitexin on cytoskeletal proteins in RBMECs after OGD (Fig. 3d, e). Representative confocal images of RBMECs showed F-actin cytoskeleton staining with FITC-phalloidin. In the control group, phalloidin staining in RBMECs demonstrated no obvious stress fiber formation, which appeared as large bundles of actin filaments. Instead, the cells showed an apparent increase in stress fiber formation in the OGD group. Treatment of OGD-exposed cells with bumetanide (100 mM; 24 h) or vitexin (100 mM; 24 h) resulted in a decrease in F-actin stress fiber formation (Fig. 3b).

Consistent with the above findings, western blot analysis of lysates from cultured RBMECs also proved that the significant increase in F-actin levels after OGD could be rescued by bumetanide (100 mM; 24 h) or vitexin (100 mM; 24 h) treatment (Fig. 3c). These results further validated our hypothesis that vitexin inhibits OGD-induced stress fiber formation in RBMECs through the NKCC1/F-actin pathway.

### Vitexin restored the expression of ZO-1 and alleviated BBB breakdown

**Fig. 4:**
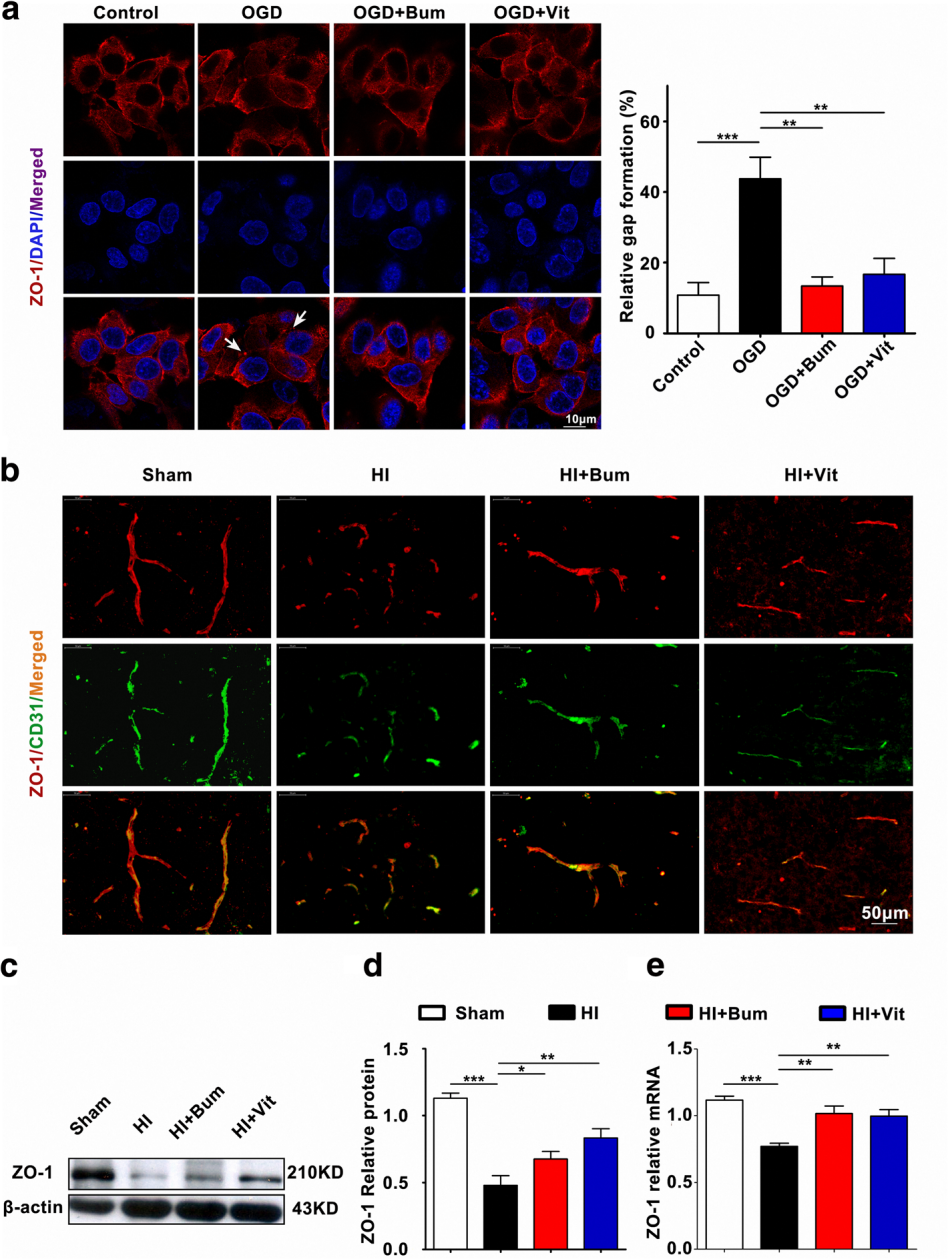
Vitexin inhibited HI-induced BBB destruction assayed by tight junction-related ZO-1. **a** TJs are characteristically located at cell–cell contact sites and are intact under physiological conditions. Confocal image of ZO-1 demonstrated disruption of the tight junctions and gap formation following OGD in RBMECs. Arrows indicate tight junction disruption. Scale bar = 50 μm. **b** Immunofluorescence staining for ZO-1 (red) and CD31 (green), a capillary endothelia marker, in the ischemic cortex of the sham, HI, HI+Bum, and HI+Vit groups 24 h after HI. Merged images of ZO-1 and CD31 staining are also shown. Scale bar = 50 μm. **c**, **d** Representative western blot for ZO-1 protein levels in the cerebral cortex from rats of each group. Densitometric value of the protein bands normalized to the respective β-actin is also shown. *P < 0.05, **P < 0.01, ***P < 0.001. **e** The mRNA expression of ZO-1 in the ipsi-ischemic brain tissue of each group was analyzed by real-time quantitative PCR. Data are shown as the mean ± SEM; **P < 0.01, ***P < 0.001 in comparison with the HI group, *n* = 4~6 per group, based on a one-way ANOVA

Robust actin polymerization and stress fiber formation in RBMECs lead to cell contraction and redistribution/disassembly of tight-junction proteins (TJs) [48]. Confocal imaging demonstrated that OGD weakened ZO-1 expression at extracellular cell–cell contact sites (Fig. 4a). In the control group, the subcellular location of ZO-1 was presented continuously at the RBMEC membrane, outlining the points of cell–cell contact and presumably the TJs. When cells were exposed to OGD, ZO-1 showed a discontinuous and diffuse pattern of staining at regions of cell–cell contact (Fig. 4a). Compared with the OGD group, those treated with vitexin exhibited more areas where the membrane-bound location of ZO-1 was maintained (Fig. 4a).

Alteration in TJ-associated proteins such as ZO-1 has been reported to contribute to the loss of BBB function in many CNS diseases and disorders [56]. Next, ZO-1 expression in the brain tissue from animals of each group was detected by immunofluorescence staining. ZO-1 expression was greatly reduced in CD31-positive capillaries in the ischemic penumbra region, which is indicative of a damaged BBB (Fig. 4b). However, after treatment with bumetanide or vitexin, a certain degree of rescue of ZO-1 expression was observed, suggesting that BBB destruction after HI was attenuated. Similarly, western blotting and q-PCR also proved that the significant reduction in ZO-1 expression after HI could be rescued by vitexin treatment (Fig. 4c, d). Based on these experiments, we concluded that vitexin protected ischemic brain damage through increasing ZO-1 expression, which consequently improved the integrity of the BBB and protected vasogenic edema.

### Vitexin reduces hypoxia-ischemia-induced neutrophil infiltration and IL-1β, IL-6, and TNF expression

**Fig. 5:**
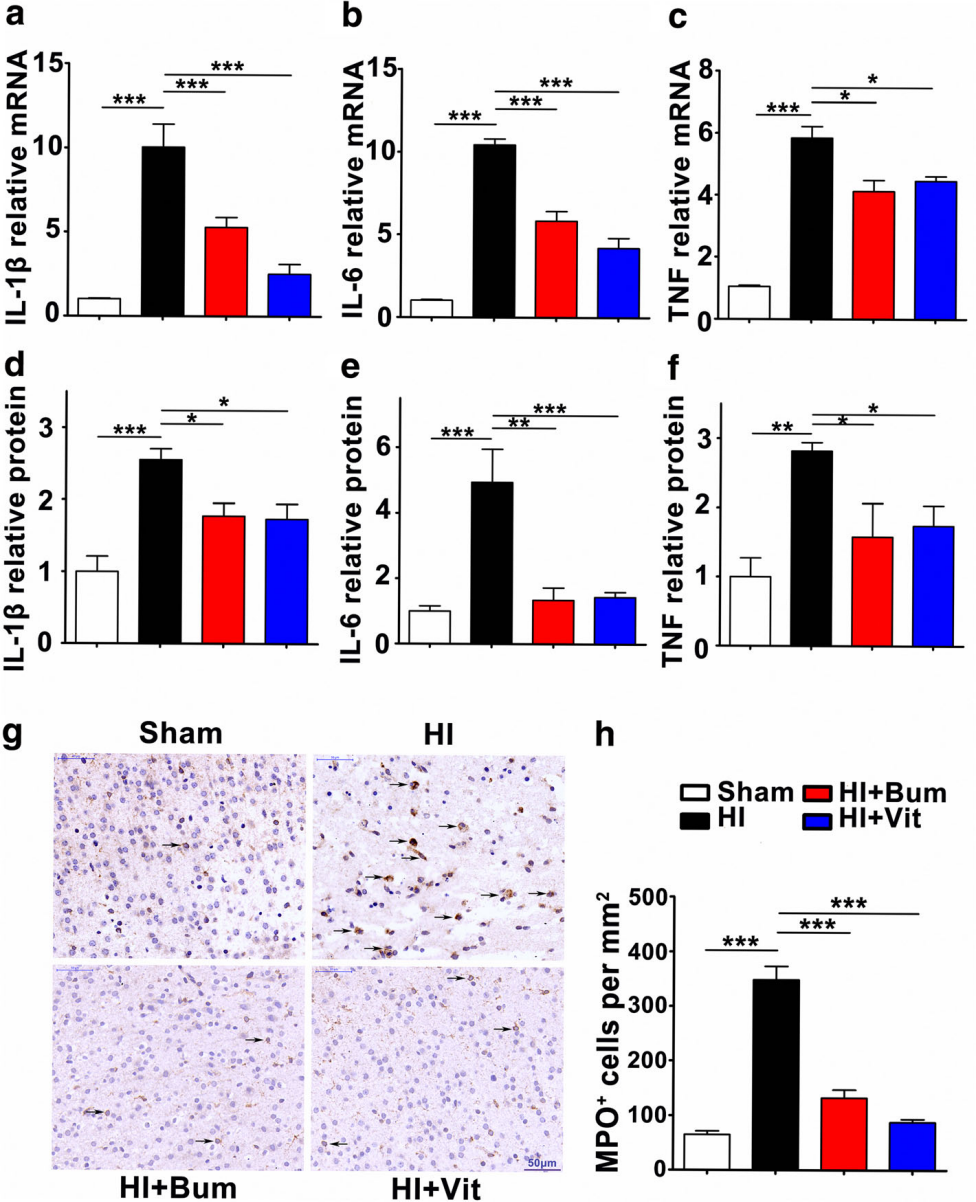
Vitexin alleviated HI-induced neutrophil infiltration and inflammatory cytokine expression. Panels **a**, **b**, and **c** show the graphical representation of the fold changes in IL-1β, IL-6, and TNF mRNA, respectively. Relative protein levels of IL-1β (**d**), IL-6 (**e**), and TNF (**f**) in brain tissue samples were measured with ELISA. Data were normalized against the protein level of the sham group, from six separate experiments. **g** Cortical penumbral regions of coronal sections from the rats of each group were subjected to immunohistochemistry using an anti-MPO antibody, and stereological counts of MPO^+^ cells (arrows) in each group are shown. Scale bar = 50 μm. **h** The graph shows the mean number of MPO^+^ cells per square millimeter. Data are expressed as the mean ± SEM; one-way ANOVA, *P < 0.05, **P < 0.01, ***P < 0.001 in comparison with the HI group, *n* = 5 per group

Mounting evidence suggests that inflammation is a key contributor to the severity of CNS hypoxia-ischemia injury. In rats subjected to 3.5 h of HI, their ipsilateral hemispheres displayed an inflammatory response as shown by increased expression of hallmark cytokines such as IL-1β, IL-6, and TNF. We further evaluated changes in the mRNA and protein levels of pro-inflammatory cytokine at 24 h after HI insult by q-PCR and ELISA. We demonstrated that HI caused a significant increasement (***P < 0.001) in the secretion of IL-1β, IL-6, and TNF when compared to sham treatment. However, treatment with vitexin (45 mg/kg) reduced IL-β, IL-6, and TNF mRNA at 24 h after HI (Fig. 5a–c, *P < 0.05, ***P < 0.001). Correspondingly, a significant decrease of protein levels of IL-β, IL-6, and TNF had also been detected (Fig. 5d–f). In sum, these results illustrate that vitexin is a potent suppressor of HI-induced inflammation. To investigate the effect of vitexin on neutrophil infiltration into ipsilateral hemispheres at 24 h after HI, we performed immunohistochemistry of coronal sections from the ipsilateral cortex to detect MPO^+^ cells using anti-MPO antibodies. MPO activity is an indicator of inflammation and is used to evaluate neutrophil accumulation [57]. Neutrophils were rarely observed in the sham-operated rats, whereas they were more abundant and more extensively distributed in the HI group, particularly in the ischemic penumbra (Fig. 5g, h). After treatment with bumetanide or vitexin, the number of MPO^+^ cells in the ipsilateral penumbra was dramatically reduced compared with that in the HI group (Fig. 5g, h). Collectively, these findings strongly suggest that vitexin can reduce the infiltration of neutrophils to a large extent under HI conditions.

### Effects of vitexin on the spontaneous EEG seizures

**Fig. 6:**
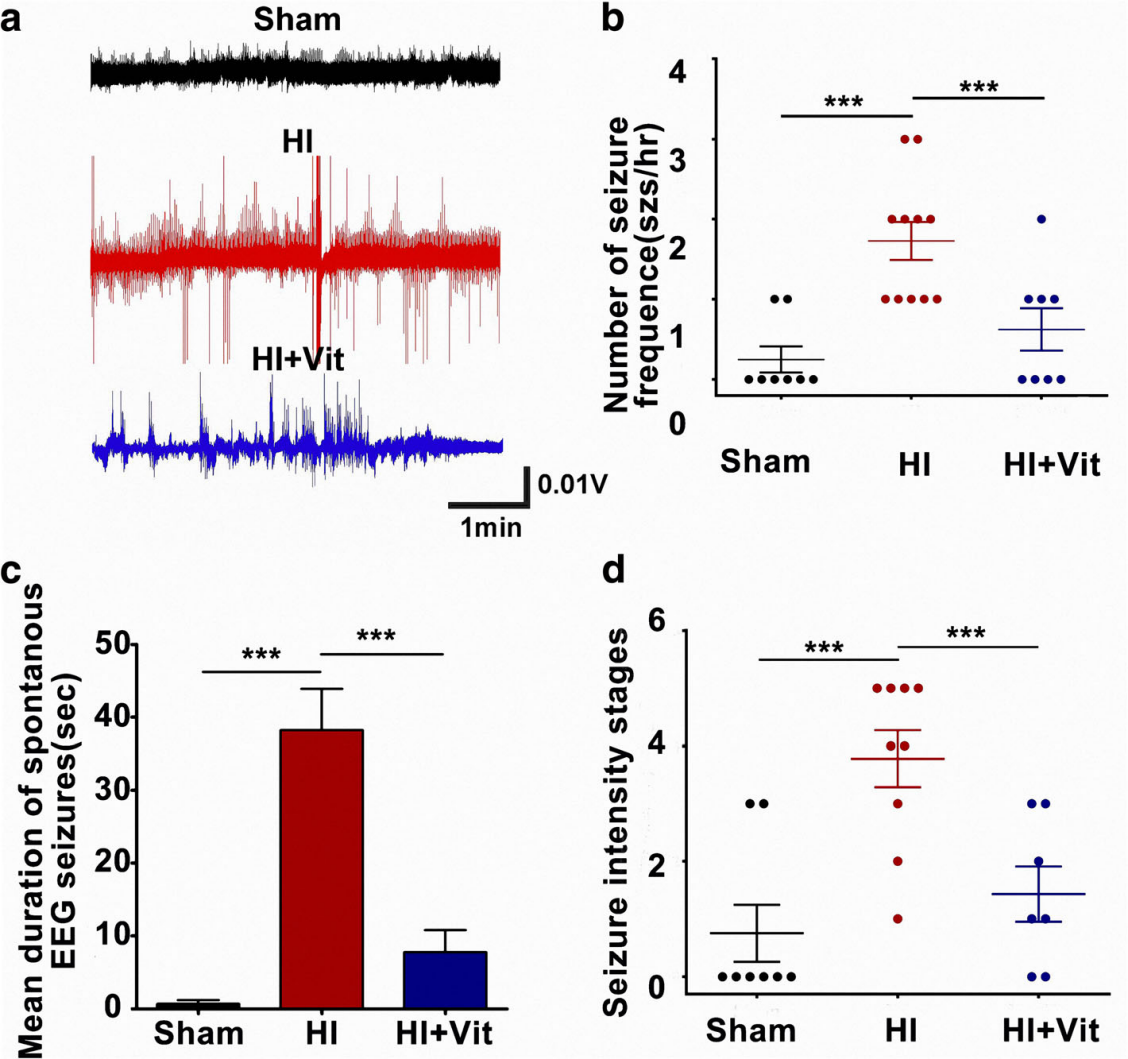
Vitexin suppressed spontaneous EEG seizures following hypoxia-induced neonatal seizures in vivo. **a** Representative traces of electroencephalograph (EEG) recordings from the sham (top, black trace), HI (middle, red trace), and HI+Vit (bottom, blue trace) groups. **b** The histograms demonstrate the seizure intensity stage of the sham, HI, and vitexin-treated groups incited after 40 mg/kg PTZ treatment. Vitexin treatment after HI showed a strong tendency to decrease the mean duration and frequency of seizure events in rats after hypoxia-induced seizures in 2-h recording sessions (**c**, **d**). *n* = 8~ 11 rats per experimental group. **P < 0.01, ***P < 0.001; one-way ANOVA with the Newman-Keuls test

Based on a previous study, we provided evidence that hypoxia-ischemia induced neonatal seizure which is thought to contribute to abnormal brain activity [51]. We therefore performed EEG monitoring to examine whether vitexin treatment in the early phase after HI has a long-term effect on HI rats. Rats were sorted into a sham group, HI group, or vitexin treatment group at P30 (Fig. 1a). The P7 rats were treated with HI and were then injected with vitexin (45 mg/kg, 5 min after HI). Electrodes were implanted in rats (*n* = 15 per group) on P28, and after 3 days of recovery, spontaneous EEG seizures were recorded in freely moving animals (Fig. 6a). During the EEG recording session, no spontaneous EEG seizures were observed in rats in the sham group. Rats in the HI+Vit group, which were given an intraperitoneal injection of vitexin, exhibited less intense spontaneous EEG seizures than rats in the HI group. Vitexin-treated animals also exhibited a marked reduction in the mean duration of spontaneous EEG seizures from 38.22 s/seizures (min. 14.0 s/seizures; max. 61.0 s/seizures) in the HI group to 7.75 s/seizures (min. 5.0 s/seizures; max. 21.0 s/seizures) in the HI+Vit group (Fig. 6b). Also, vitexin treatment significantly decreased the frequency of spontaneous EEG seizures in rats in the HI+Vit group (0.62 (min. 0; max. 2)) compared with the frequency in rats in the HI group (1.72 (min. 1; max. 3); Fig. 6b; *P < 0.05). Taken together, our results demonstrated that treatment with vitexin during a vulnerable period was able to decrease pentylenetetrazol (PTZ)-induced seizure in rats after HI.

## Discussion

In this research, we revealed that vitexin could be an effective neuroprotective agent to reduce the severity of seizures in hypoxic-ischemic rat model. Such neuroprotective effects were potentially mediated through inhibition of NKCC1/F-actin expression, which subsequently improved the tight junctions and, therefore, the integrity of the BBB and reduced infiltration of neutrophils. In summary, our data showed a previously unexplored mechanism by which vitexin exerts its neuroprotective effect after HI-induced injury through diminished NKCC1 expression.

**Fig. 7:**
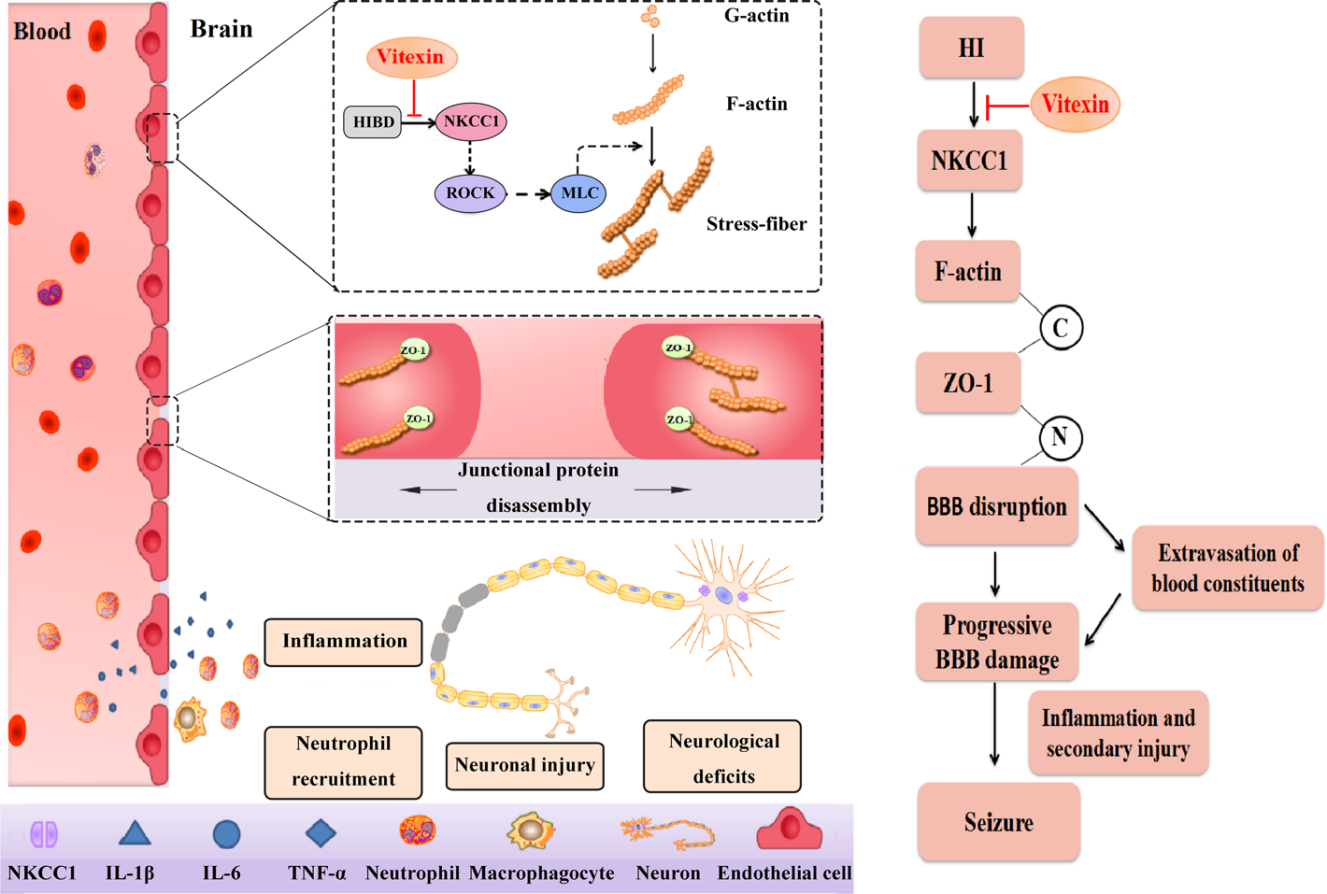
Schematic diagram depicting the proposed mechanism involved HI-induced seizure. The evolution of seizures after HI progresses along the following steps: (1) NKCC1 expression is enhanced in RBMECs under HI, which appears to affect cytoskeletal alterations. Actin polymerization is enhanced and F-actin^+^ stress fibers are formed inside injured RBMECs. (2) Stress fiber formation causes endothelial contraction and TJs (for example, ZO-1). (3) The disassembly and redistribution of TJs lead to subtle BBB hyperpermeability and induce the recruitment of neutrophils into ischemic regions, at least in part through increased production of neutrophil chemoattractant. (4) Aberrant increase in neutrophil infiltration causes abnormal inflammation and subsequent pathological events in the brain. (5) As a result, peripheral leukocyte infiltration leads to exacerbation of inflammation and neuronal injury, which results in epilepsy and eventually seizures. By targeting the early NKCC1 upregulation, vitexin attenuates BBB disruption at the start, as well as subsequent tissue injury, thereby offering long-term functional improvements

Flavonoids are polyphenolic structures that are naturally present in most plants and consumed daily with no adverse effects reported. Of interest, flavonoids and their glycosides have been shown to exert mild to potent activity in several seizure and epilepsy animal models [58, 59]. Investigations of the underlying mechanism of action have led to the assumption that these structures allosterically modulate GABA_A_ receptors by binding to the benzodiazepine receptor site [46]. Thus, vitexin seems to be a ligand for benzodiazepine receptors [44–46]. To the best of our knowledge, there has been no report on the antiepileptic effect of vitexin in an HI-induced epileptic rat model (Fig. 7).

Many of the available anti-seizure drugs block seizures by enhancing inhibitory GABA_A_ receptor activity in the brain. The inhibitory activity of GABA_A_ receptor activation depends on low [Cl^−^]_*i*_, which is modulated by the opposing regulation of NKCC1 and KCC2 in neurons [60, 61]. NKCC1 is frequently expressed in developing CNS regions, and its activity has been associated with the depolarizing actions of GABA [62, 63]. The increase in NKCC1 levels has been shown to be involved in neonatal seizures [64, 65]. Inappropriate activation and increased expression of NKCC1 will contribute to increased [Cl^−^]_*i*_, which in turn renders GABAergic input less inhibitory and more seizure-prone. Thus, we sought drugs targeting NKCC1 to reduce seizures by lowering [Cl^−^]_*i*_. Taken together, our results are the first to reveal that vitexin disturbs the expression of NKCC1 in vivo and in vitro, which may subsequently protect the brain against seizures.

With regard to the fact that seizures are a process that includes BBB dysfunction and neuronal death, we aimed to investigate the protective role of vitexin in seizures. A previous study on other flavonoids proved its neuroprotective effect after SAH in rats through upregulating the expression of ZO-1 and occludin following subarachnoid hemorrhage (SAH), which was closely related to the integrity of the BBB [28]. Injuries such as ischemia and traumatic brain injury lead to a disruption and reconstruction of ZO-1 and occludin and an increase in BBB permeability [57]. Studies have demonstrated that hypoxia mainly influences the distribution of ZO-1, resulting in a disruption of TJ proteins at cell–cell contacts and an increase in endothelial cell permeability [58]. A reduction in BBB permeability alleviates cerebral ischemia injury in both transient and permanent cerebral ischemia [57, 59, 60]. As our previous study has shown, vitexin attenuated neuronal cell death and brain edema and preserved BBB disruption after neonatal HI in a rat pup model [37]. Further research is needed to evaluate the molecular mechanism underlying BBB preservation by vitexin.

Furthermore, flavonoids are also known to exert potent anti-inflammatory effects in the brain [66] and directly modulate key components of the inflammatory cascade [67]. Concomitantly, the heightened expression of the pro-inflammatory cytokines such as IL-1β, IL-6, TNF, and neutrophils was significantly attenuated by vitexin treatment in the HI-stimulated brain, further demonstrating diminished inflammation by this agent (Fig. 5). Our results are in partial agreement with the results of Borghi et al., which demonstrated an analgesic effect of vitexin through reducing TNF-α and IL-1β expression in mice [68]. In addition to increasing inflammatory cytokines, neutrophil infiltration also induces endothelial injury and increases BBB permeability [69].

A previous study concluded that NKCC1 not only regulates migration but also alters the actin cytoskeleton, the migratory engine of cells [19, 65]. Therefore, the relationship between NKCC1 and actin appears to be important for understanding NKCC1 functions [66]. In an effort to dissect NKCC1’s role in actin regulation, we use confocal imaging to study NKCC1 protein-interacting partners. We found that NKCC1 interacts with F-actin, which plays a key role in actin polymerization/depolymerization in RBMECs (Fig. 3a, b). The disruption of the BBB and the increase in permeability were related to increased F-actin and enhanced stress fiber formation during hypoxia/reoxygenation [29, 70–72]. In summary, we found that inhibition of NKCC1 not only controls the Cl^−^ concentration but also decreases the formation of stress fibers through regulating the actin cytoskeleton, which eventually leads to the destruction of the BBB.

In summary, our data revealed a previously unexplored neuroprotective effect of vitexin through inhibition of the NKCC1/F-actin pathway, subsequently ameliorating the BBB collapse and inflammatory responses in the context of HIBD. In addition, another potential mechanism by which vitexin inhibits the onset of epilepsy may be the direct inhibition of the occurrence of neutrophil infiltration. Therefore, with these specific properties, vitexin plays a vital role in preventing various diseases associated with NKCC1 upregulation, such as cardiovascular, glioblastoma, stroke, and neurodegenerative disease.

## Conclusions

Collectively, our results and results of previous study strongly indicate that NKCC1 is a vital contributor to secondary damage after HI in rats. Vitexin is beneficial in the treatment of ischemic insult, which potentially occurs via disturbances in the NKCC1/F-actin signaling pathways, alleviating the severity of BBB collapse, controlling inflammation, and improving neurological recovery after HI. Vitexin is a widely used drug with few adverse effects, and the utilization of this long-established drug for a new use may be a promising way to develop an effective therapy for HIBD-induced epilepsy.

## Abbreviations

BBB: Blood-brain-barrier
DAPI: 4:6-diamidino-2-phenylindole
EEG: Electroencephalography
HIBD: Hypoxia-ischemia brain damage
HIE: Hypoxic-ischemic encephalopathy
IL-1β: Interleukin 1β
IL-6: Interleukin 6
NKCC1: Na^+^-K^+^-Cl^−^ co-transporter1
OGD: Oxygen glucose deprivation
PBS: Phosphate-buffered saline
RBMECs: Rat brain microvascular endothelial cells
TJ: Tight-junction protein
TNF: Tumor necrosis factor
ZO-1: Zona occludens-1
